# Impact of co-presence of malnutrition-inflammation-atherosclerosis factors on prognosis in lower extremity artery disease patients after endovascular therapy

**DOI:** 10.1007/s12928-024-01058-6

**Published:** 2024-10-24

**Authors:** Kenta Ohmure, Daisuke Kanda, Yoshiyuki Ikeda, Akihiro Tokushige, Takeshi Sonoda, Ryo Arikawa, Kazuhiro Anzaki, Mitsuru Ohishi

**Affiliations:** Department of Cardiovascular Medicine and Hypertension, Graduate School of Medical and Dental Sciences, Kagoshima University, 8-35-1 Sakuragaoka, Kagoshima, Kagoshima 890-8520 Japan

**Keywords:** Lower extremity artery disease, Critical limb-threatening ischemia, Malnutrition, Inflammation, Atherosclerosis

## Abstract

Malnutrition and inflammation are common problems of adverse outcomes in various diseases as atherosclerosis. Recently, the concept of malnutrition-inflammation-atherosclerosis (MIA) syndrome is known to a crucial prognostic factor in patients with end-stage renal disease. We investigated the prognostic impact of the co-presence of MIA factors in patients with lower-extremity artery disease (LEAD) after endovascular therapy (EVT). This retrospective study included 284 patients with LEAD who underwent EVT. MIA factors were defined in patients with: low geriatric nutritional risk index (< 92) as “malnourished”; greater high-sensitivity C-reactive protein levels (≥ 0.1 mg/dL) as “inflamed”; a history of coronary artery revascularization, lacunar or atherothrombotic brain infarction as “atherosclerotic”. We examined the relationship between baseline characteristics and major adverse cardiovascular and cerebrovascular events (MACCE) including all-cause mortality. Sixty-two patients (22%) died and 72 patients (25%) had MACCE, including all-cause death. Forty-four (16%) patients had every 3 of MIA (3-MIA) factors. The mean follow-up period was 737 days. Multivariate Cox proportional hazards regression analysis models revealed that 3-MIA factors correlated significantly with all-cause death [hazard ratio (HR) 3.46, 95% confidence interval (CI) 1.89–6.34; *p* < 0.001] and MACCE (HR 2.91, 95% CI 1.62–5.22; *p* < 0.001), after adjusting for relevant factors. Kaplan–Meier analysis revealed that the rates of all-cause death (*p* < 0.001) and MACCE (*p* < 0.001) were significantly higher when the MIA factors overlapped and are observed in patients with chronic limb-threatening ischemia. Among patients with LEAD after EVT, the co-presence of MIA factors was an independent risk factor for all-cause death and MACCE.

**Graphical abstract:**

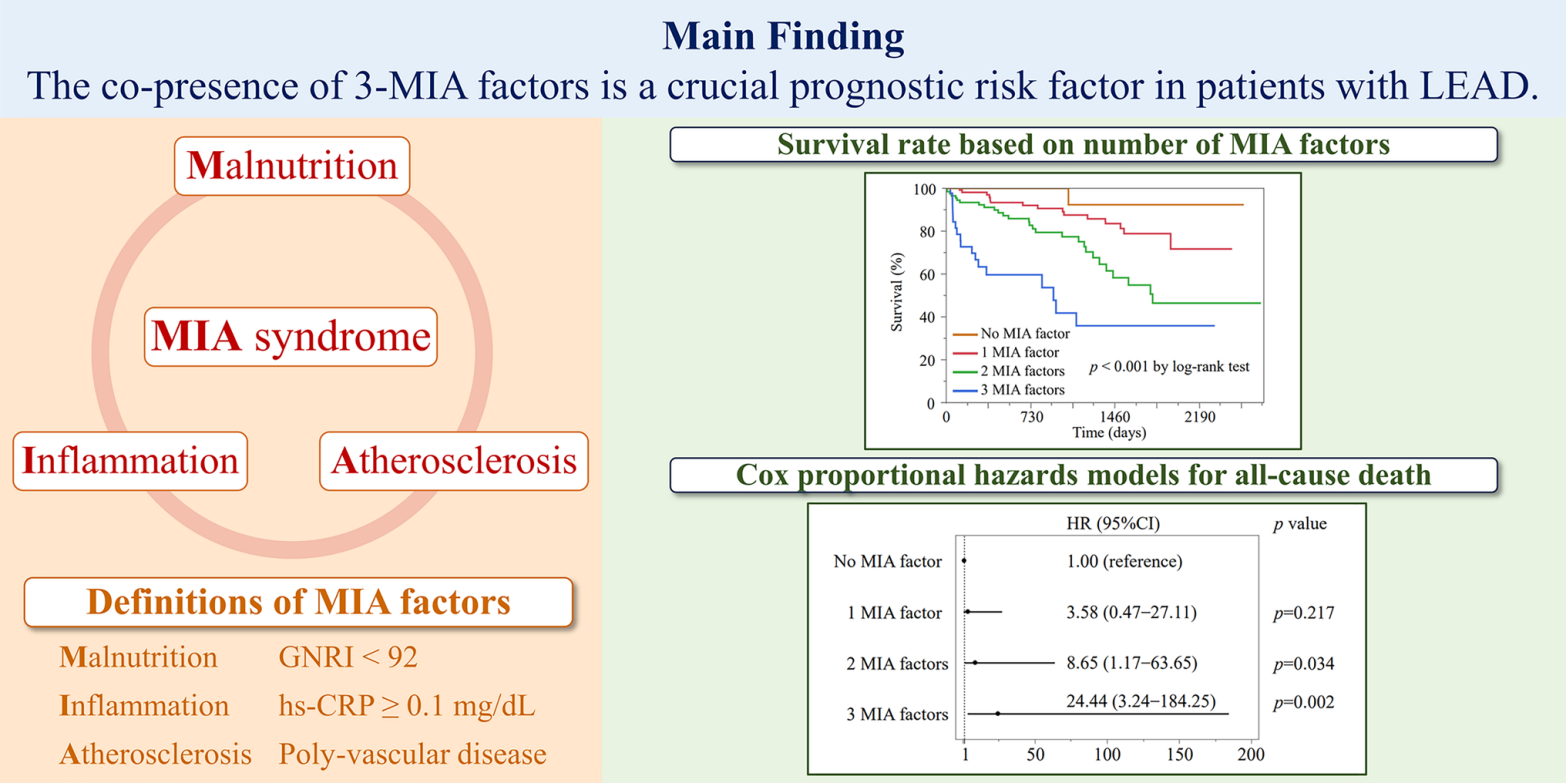

**Supplementary Information:**

The online version contains supplementary material available at 10.1007/s12928-024-01058-6.

## Introduction

Lower extremity artery disease (LEAD) is a manifestation of systemic atherosclerosis caused by the narrowing of arteries in the lower extremities. It is associated with an increased risk of cardiovascular events, functional impairment, and decreased quality of life. LEAD patients have a high risk of mortality, often co-exist with coronary artery disease (CAD) or stroke with an increased occurrence in recent years [1]. Despite advances in medical and endovascular therapy (EVT), the prognosis of LEAD patients remains poor, with CAD and cerebrovascular disease (CVD) being the most common causes of death [2].

Malnutrition is a common issue among hospitalized patients and has been reported to be associated with a worse prognosis among patients with chronic diseases, such as chronic kidney disease (CKD) [3]. Furthermore, malnutrition is associated with chronic inflammation. Malnutrition and inflammation are common problems in patients undergoing hemodialysis and are associated with adverse outcomes [4]. Inflammation plays a central role in the development and progression of atherosclerosis [5]. Additionally, several studies have examined inflammatory markers as predictors of cardiovascular events and death in CAD and LEAD including acute coronary syndrome and chronic limb-threatening ischemia (CLTI) [6, 7].

Recently, reports indicate a strong correlation between malnutrition, inflammation, and atherosclerosis as the basis of malnutrition-inflammation-atherosclerosis (MIA) syndrome which is widely accepted, especially in patients with end-stage renal disease [8]. Although several studies have reported the impact of malnutrition [9, 10], inflammation [11], and atherosclerosis [12] on the prognosis of patients with LEAD, the effect of the co-presence of these three factors on the prognosis of patients with LEAD is uncertain.

This novel study aimed to investigate the prognostic impact of the co-presence of MIA factors in patients with LEAD after EVT.

## Methods

### Study population

We conducted a retrospective and observational study at a single center consisting of 287 consecutive patients with LEAD admitted to Kagoshima University Hospital between January 2015 and February 2022 to undergo EVT of the lower extremity artery. Two patients with acute coronary syndrome who underwent EVT for insertion of mechanical circulatory support device and one patient with end-stage pancreatic cancer were excluded. Finally, 284 patients were included in this study.

This study was approved by the Research and Ethics Committee of Kagoshima University Hospital (approval number 200035疫) and conducted in accordance with the ethical principles of the 1975 Declaration of Helsinki. All patients provided written informed consent after admission, agreeing with the use of clinical test data for scientific research by the hospital.

Experienced cardiologists performed the EVT according to the Trans-Atlantic Inter-Society Consensus II guidelines [13]. Demographics, medical history, comorbidities, and laboratory data were collected from individual medical records at admission. Patients were followed up at our hospital or by their physicians. Subsequently, we examined the relationship between baseline characteristics, including MIA factors, all-cause death, and major adverse cardiovascular and cerebrovascular events (MACCE) after EVT.

### Measurements of clinical parameters and assessment of the geriatric nutritional risk index (GNRI)

Blood samples were collected upon admission to our hospital, prior to EVT. The levels of high-sensitivity C-reactive protein (hs-CRP), triglyceride, high-density lipoprotein cholesterol, low-density lipoprotein cholesterol (LDL-C), and serum albumin were measured. The estimated glomerular filtration rate (eGFR) was calculated using the Modification of Diet in Renal Disease equation with coefficients modified for Japanese patients as follows: eGFR (mL/min/1.73 m^2^) = 194 × serum creatinine (mg/dL)^−1.094^ × age (years)^−0.287^ (× 0.739 for female participants) [14]. In addition, echocardiograms, including the left ventricular ejection fraction (LVEF), were performed on admission.

This study assessed the nutritional status using the GNRI, which was calculated using the following equation: GNRI = 14.89 × serum albumin level in g/dL + 41.7 × (body weight in kg/ideal body weight in kg) [15]. The body weight/ideal body weight ratio was set to 1 when the body weight of the patient exceeded the ideal body weight. Ideal body weight was calculated using a body mass index (BMI) of 22 kg/m^2^. BMI was computed as body weight divided by height squared (kg/m^2^) [15].

### Definitions

LEAD was diagnosed based on an ankle-brachial index < 0.9 and lower extremity artery stenosis, or occlusion on imaging modalities such as ultrasonography, contrast-enhanced computed tomography, or catheter angiography [13]. CLTI was defined by the presence of ischemic rest pain, with or without tissue loss (ulcers, gangrene) or infection, and lower extremity ischemia was defined as ankle joint pressure < 50 mmHg or SPP < 30 mmHg [16].

Current smokers were defined as patients who had smoked at least 100 cigarettes in their lifetime and currently smoked cigarettes at the time of admission. The antiplatelet drugs included aspirin, clopidogrel, ticlopidine, and cilostazol.

The MIA syndrome is characterized by malnutrition, inflammation, and atherosclerosis. These three factors are defined as follows: patients with low GNRI (< 92) at baseline were defined as “malnourished” based on previously published thresholds [15]. Furthermore, patients with high hs-CRP levels (≥ 0.1 mg/dL) at baseline were defined as “inflamed” based on guidelines from the Centers for Disease Control and Prevention and the American Heart Association [17]. Patients who underwent percutaneous coronary interventions or coronary artery bypass grafting for CAD or with a history of lacunar stroke or atherothrombotic brain infarction were defined as “atherosclerotic.”

### Clinical outcomes

Clinical outcomes were retrospectively collected during the follow-up. All-cause mortality was defined as death after EVT. MACCE constitutes a composite endpoint, including all-cause death, nonfatal myocardial infarction, and stroke.

### Statistical analyses

Descriptive statistics are presented as frequency (percentage) for categorical variables and mean ± standard deviation or median and interquartile range (IQR) for continuous variables. Fisher’s exact test was utilized in comparing the incidence of categorical variables, which were expressed as frequencies and percentages. Continuous variables were compared between the survivor group (SG) and deceased group (DG) using Student’s *t-*test (for values showing a normal distribution) or the Wilcoxon rank-sum test (for values showing a non-normal distribution). Cox proportional hazards regression analysis was employed in analyzing factors associated with all-cause death and MACCE, reporting hazard ratios (HRs) and 95% confidence intervals (CIs), and results based on MIA factors were expressed using forest plots. Independent associations between all-cause death or MACCE and baseline characteristics were assessed by multivariate Cox proportional hazards model analysis using relevant factors, defined as variables with *p* < 0.05 on univariate analysis. The cumulative survival rate and incidence of MACCE based on MIA factors were estimated using a Kaplan–Meier curve evaluated by log-rank testing. Variables with *p* < 0.05 on univariable analysis were entered into a multivariable Cox proportional hazards regression model to assess HRs for all-cause death and MACCE. The level of significance was set at *p* < 0.05. Statistical analyses were performed using the SAS software (JMP version 14.0).

## Results

### Baseline characteristics

The baseline clinical characteristics of the patients are shown in Table 1. The mean age was 73 ± 11 years, and 191 patients (67%) were men. Notable comorbidities included hypertension (86%), diabetes mellitus (62%), dyslipidemia (67%), CKD (76%), and hemodialysis (36%). Of the 284 patients, 44 (16%) had 3-MIA factors.

**Table 1:** Comparison of patient characteristics between the survivor and deceased groups

Variables	Over all	Survivor group	Deceased group	*p* value
	(n = 284)	(n = 222)	(n = 62)	
Age, y	73 ± 11	72 ± 11	75 ± 10	0.093
Sex: male, n (%)	191 (67)	142 (64)	49 (79)	0.032
BMI, kg/m^2^	22.1 [19.9, 24.2]	22.3 [20.3, 24.7]	21.4 [18.9, 22.9]	0.003
Risk factors, n (%)				
Hypertension	245 (86)	191 (86)	54 (87)	1
Diabetes mellitus	176 (62)	135 (61)	41 (66)	0.464
Dyslipidemia	191 (67)	157 (71)	34 (55)	0.022
Current smoking	51 (18)	42 (19)	9 (15)	0.574
CKD	217 (76)	161 (73)	56 (90)	0.004
Hemodialysis	102 (36)	63 (28)	39 (63)	< 0.001
Medications, n (%)				
CCB	155 (55)	124 (56)	31 (50)	0.471
RAASi	147 (52)	118 (53)	29 (47)	0.392
β-blocker	88 (31)	69 (31)	19 (31)	1
Antiplatelet drug	230 (84)	181 (81)	49 (79)	0.715
Statin	151 (53)	125 (56)	26 (42)	0.061
Laboratory data				
hs-CRP, mg/dL	0.26 [0.09, 1.17]	0.22 [0.08, 1.15]	0.47 [0.17, 1.31]	0.017
Total cholesterol, mg/dL	162 [138, 190]	163 [137, 189]	155 [139, 192]	0.788
Triglyceride, mg/dL	111 [81, 154]	112 [83, 158]	97 [75, 124]	0.022
HDL-C, mg/dL	48 [39, 58]	47 [39, 58]	49 [41, 60]	0.357
LDL-C, mg/dL	89 [71, 114]	89 [72, 115]	87 [71, 113]	0.698
Albumin, g/dL	3.7 [3.3, 4.1]	3.8 [3.4, 4.2]	3.6 [3.3, 3.9]	0.006
eGFR	42.9 [9.6, 59.9]	46.2 [14.1, 62.5]	11.0 [6.9, 40.6]	< 0.001
Others				
LVEF, %	63.7 [54.5, 70.1]	63.7 [55.0, 70.8]	63.7 [53.6, 68.1]	0.259
GNRI	97.2 ± 12.5	98.6 ± 12.6	92.1 ± 10.5	< 0.001
CLTI, n (%)	141 (50)	102 (46)	39 (63)	0.022
Malnourished, n (%)	90 (32)	62 (28)	28 (45)	0.013
Inflamed, n (%)	204 (72)	151 (68)	53 (86)	0.007
Atherosclerotic, n (%)	171 (60)	127 (57)	44 (71)	0.057
3-MIA, n (%)	44 (16)	26 (12)	18 (29)	0.002

Values are presented as mean ± standard deviation or median with an interquartile range

*BMI* body mass index, *CCB* calcium channel blocker, *CKD* chronic kidney disease, *CLTI* chronic limb-threatening ischemia, *hs-CRP* high-sensitivity C-reactive protein, *eGFR* estimated glomerular filtration rate, *GNRI* geriatric nutritional risk index, *HDL-C* high-density lipoprotein cholesterol, *LDL-C* low-density lipoprotein cholesterol, *LVEF* left ventricular ejection fraction, *RAASi* renin–angiotensin–aldosterone system inhibitor, *3-MIA* co-presence of three factors of malnutrition-inflammation-atherosclerosis syndrome (malnutrition, inflammation and atherosclerosis)

### Patient characteristics according to survival status

Table 1 indicates a comparison of patient characteristics between the SG and DG. Sex(male), dyslipidemia, CKD, and hemodialysis were significantly different between the SG and DG (64% vs. 79%, *p* = 0.032; 71% vs. 55%, *p* = 0.022; 73% vs. 90%, *p* = 0.004; and 28% vs. 63%, *p* < 0.001, respectively). The SG indicated a higher BMI (median, 22.3 kg/m^2^; IQR, 20.3–24.7 kg/m^2^) than DG (median, 21.4 kg/m^2^; IQR, 18.9–22.9 kg/m^2^; *p* = 0.003).

There were no significant differences in age, hypertension, or diabetes mellitus between the two groups. The median concentration of hs-CRP was higher in the DG (median, 0.47 mg/dL; IQR, 0.17–1.31 mg/dL) than in the SG (median, 0.22 mg/dL; IQR, 0.08–1.15 mg/dL; *p* = 0.017). The GNRI was significantly lower in the DG than in the SG (92.1 ± 10.5 vs. 98.6 ± 12.6; *p* < 0.001). Moreover, the proportion of patients with CLTI or 3-MIA was significantly higher in the DG than in the SG (63% vs. 46%, *p* = 0.022, and 29% vs. 12%, *p* = 0.002, respectively).

### Clinical outcomes after EVT

The mean follow-up duration was 737 days (IQR, 271–1388) days. Sixty-two patients (22%) died after EVT, and 72 patients (25%) had MACCE, including all-cause death. The details of the 62 deaths were as follows: cardiovascular events, including two acute myocardial infarctions (14 patients); infection (21 patients); malignancy (7 patients); others (20 patients).

### Association of baseline systemic factors with all-cause death and MACCE

Cox proportional hazards regression analysis was performed to investigate the association of baseline characteristics, including 3-MIA factors, with all-cause death and MACCE. In univariate analysis, malnourished (all-cause death, HR 3.93, 95% CI 2.34–6.60, *p* < 0.001; and MACCE, HR 3.28, 95% CI 2.02–5.32, *p* < 0.001), inflamed (all-cause death, HR 3.44, 95% CI 1.69–6.98, *p* < 0.001; and MACCE, HR 2.94, 95% CI 1.58–5.47, *p* = 0.046) and 3-MIA factors (all-cause death, HR 4.59, 95% CI 2.62–8.04, *p* < 0.001; and MACCE, HR 3.89, 95% CI 2.28–6.65, *p* < 0.001) were significantly associated with both all-cause death and MACCE. Furthermore, atherosclerotic (HR 1.69, 95% CI 1.01–2.83, *p* < 0.001) showed a significant association with MACCE (Table 2). Multivariate Cox proportional hazards regression modeling revealed a strong association of 3-MIA factors with all-cause death and MACCE, even after adjusting for relevant factors, such as age, sex (male), dyslipidemia, CKD, statin, LVEF, and renin–angiotensin–aldosterone system inhibitor (all-cause death, model 3: HR 3.46, 95% CI 1.89–6.34, *p* < 0.001; and MACCE, model 4: HR 2.91, 95% CI 1.62–5.22, *p* < 0.001, respectively) (Table 3). A comparison of p-value for interaction between 3-MIA and CKD, 3-MIA and dyslipidemia were *p* = 0.824 and *p* = 0.085, respectively.

**Table 2:** Univariate Cox proportional hazard analysis for all-cause death and MACCE

Variables	All-cause death			MACCE		
	HR	95% CI	*p* value	HR	95% CI	*p* value
Age	1.04	1.02–1.07	0.002	1.03	1.02–1.05	0.018
Sex: male	1.81	0.98–3.34	0.056	1.56	0.90–2.68	0.111
Hypertension	0.93	0.44–1.96	0.85	1.14	0.54–2.37	0.734
Diabetes mellitus	1.29	0.76–2.18	0.349	1.24	0.76–2.02	0.38
Dyslipidemia	0.44	0.26–0.73	0.001	0.43	0.27–0.69	< 0.001
Current smoking	0.82	0.41–1.67	0.589	1.06	0.58–1.94	0.838
CKD	3.66	1.58–8.51	0.003	3.21	1.54–6.70	0.002
CCB	0.72	0.43–1.18	0.191	0.74	0.46–1.17	0.196
RAASi	0.65	0.40–1.08	0.095	0.62	0.39–0.99	0.045
β-blocker	0.97	0.57–1.67	0.919	0.83	0.50–1.40	0.489
Antiplatelet drug	0.81	0.44–1.50	0.503	0.81	0.46–1.44	0.476
Statin	0.44	0.27–0.74	0.002	0.44	0.27–0.70	< 0.001
CLTI	2.81	1.67–4.71	< 0.001	2.56	1.59–4.13	< 0.001
Malnourished	3.93	2.34–6.60	< 0.001	3.28	2.02–5.32	< 0.001
Inflamed	3.44	1.69–6.98	< 0.001	2.94	1.58–5.47	0.046
Atherosclerotic	1.59	0.92–2.75	0.099	1.69	1.01–2.83	< 0.001
3-MIA	4.59	2.62–8.04	< 0.001	3.89	2.28–6.65	< 0.001
LVEF	0.979	0.96–0.998	0.029	0.981	0.96–0.999	0.034

*CI* confidence interval, *HR* hazard ratio, *MACCE* major adverse cardiovascular and cerebrovascular events

The other parameters are listed in Table 1

**Table 3:** Cox proportional hazards regression analysis models of 3-MIA for all-cause death and MACCE

	All-cause death			MACCE		
	HR	95% CI	*p* value	HR	95% CI	*p* value
Unadjusted	4.59	2.62–8.04	< 0.001	3.89	2.28–6.65	< 0.001
Model 1	3.52	1.92–6.48	< 0.001	3.04	1.70–5.41	< 0.001
Model 2	3.50	1.87–6.53	< 0.001	2.94	1.63–5.31	< 0.001
Model 3	3.46	1.89–6.34	< 0.001	2.98	1.68–5.30	< 0.001
Model 4	–	–	–	2.91	1.62–5.22	< 0.001

Unadjusted, 3-MIA; Model 1 is adjusted for age, sex (male), statin, and LVEF; Model 2 is adjusted for variables in Model 1 plus dyslipidemia; Model 3 is adjusted for variables in Model 1 plus CKD; Model 4 is adjusted for variables in Model 1 plus RAASi

The parameters are listed in Tables 1 and 2

Similarly, multivariate Cox proportional hazards regression models in patients with CLTI indicated that the 3-MIA factors were significantly associated with all-cause death and MACCE (Supplemental Tables 1 and 2). A comparison of p-value for interaction between 3-MIA and CKD, 3-MIA and dyslipidemia were *p* = 0.999 and *p* = 0.044, respectively.

### Associations between clinical outcomes and number of MIA factors

**Fig. 1:**
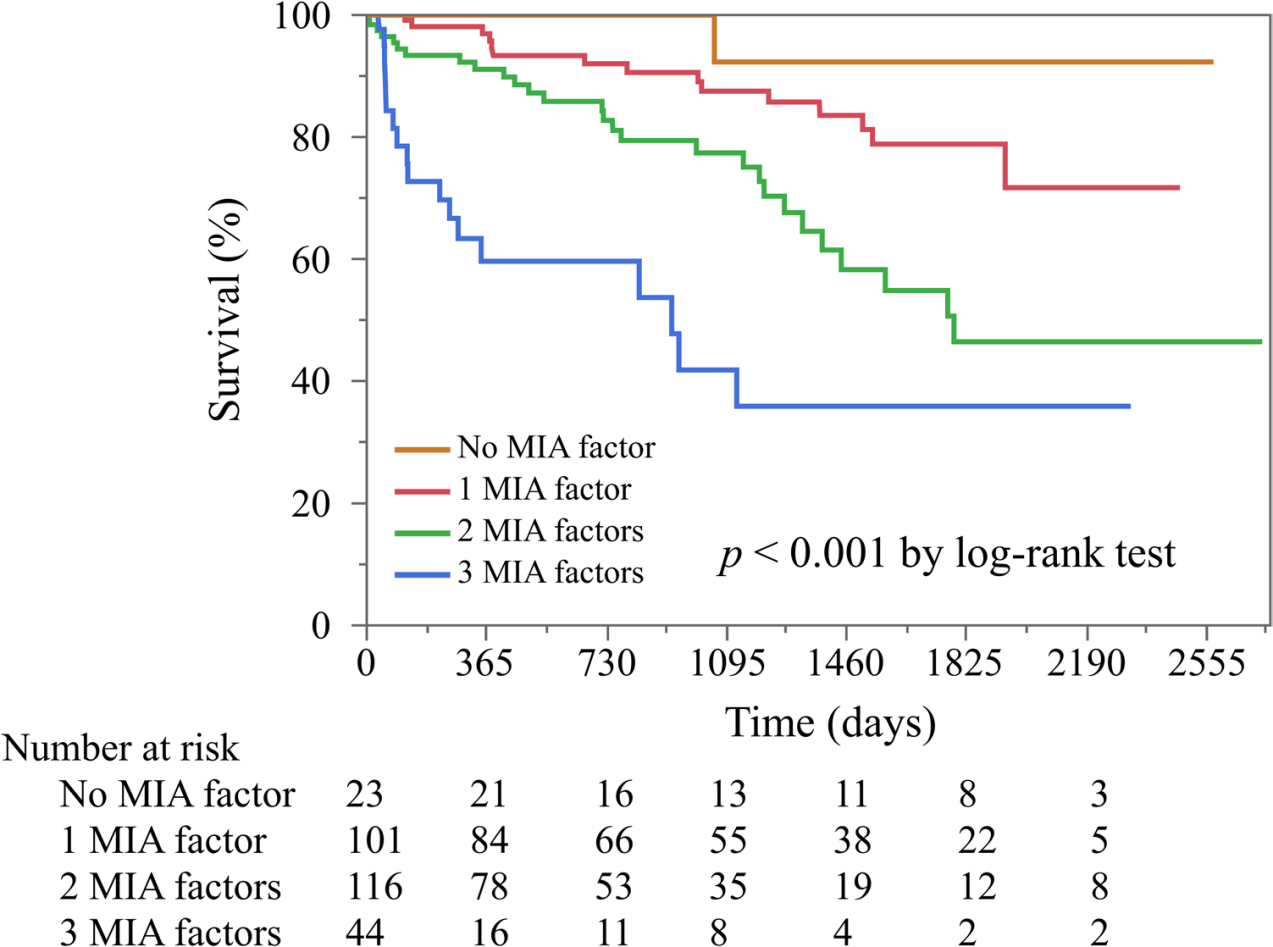
Kaplan–Meier analysis of survival rate based on number of MIA factors. *MIA factor* a factor of malnutrition-inflammation-atherosclerosis syndrome

**Fig. 2:**
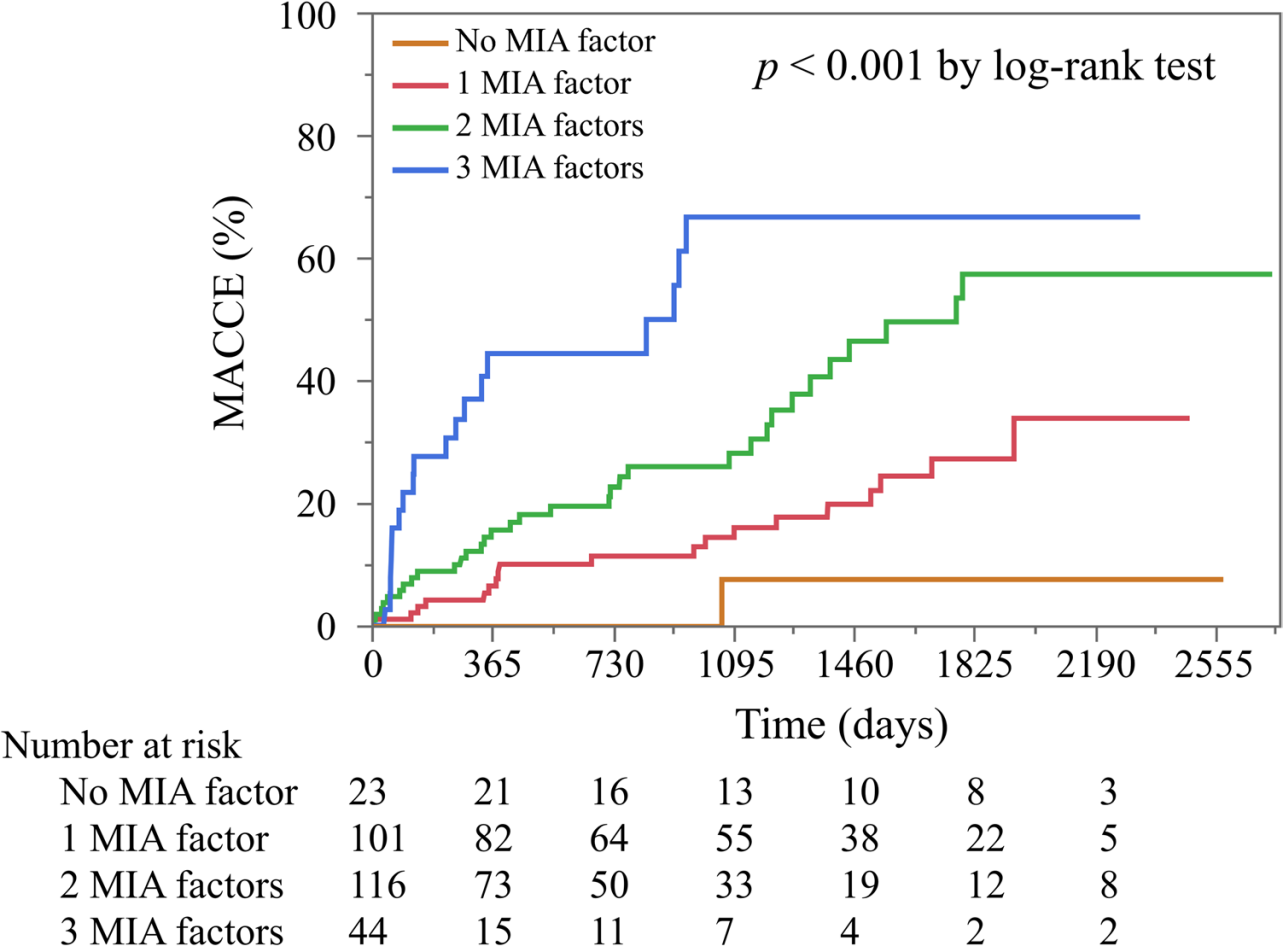
Cumulative incidences of MACCE based on number of MIA factors. *MACCE* major adverse cardiovascular and cerebrovascular events, *MIA* malnutrition-inflammation-atherosclerosis syndrome

**Fig. 3:**
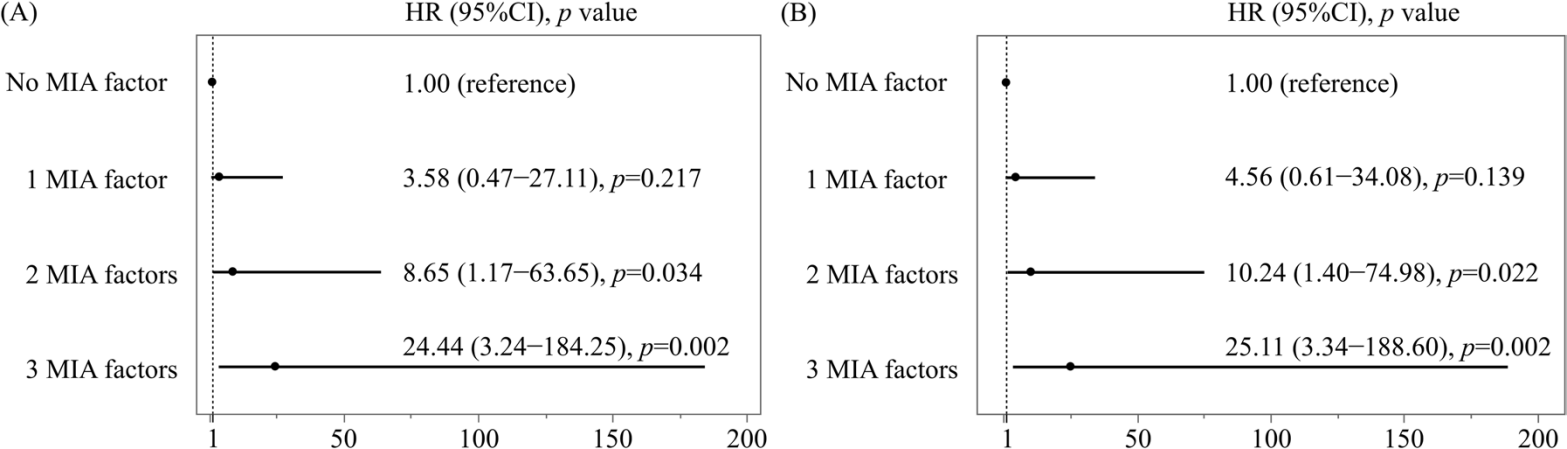
Cox proportional hazards models for all-cause death and MACCE. The forest plot shows the HR (with 95% CI) for all-cause death (**A**) and MACCE (**B**) among the number of MIA factors. *CI* confidence interval, *HR* hazard ratio, *MACCE* major adverse cardiovascular and cerebrovascular events, *MIA* malnutrition-inflammation-atherosclerosis syndrome

Kaplan–Meier analysis exhibited a significant increase in the rates of all-cause death (*p* < 0.001) and MACCE (*p* < 0.001) after EVT (Fig. 1 and 2). Moreover, as the MIA factors overlapped, the HR of all-cause death and the incidence of MACCE markedly increased (Fig. 3).

Similarly, in patients with CLTI, the HR of all-cause death and the incidence of MACCE increased. Additionally, the Kaplan–Meier analysis exhibited a significantly increased rate of all-cause death (*p* < 0.001) and MACCE (*p* < 0.001) as the number of MIA factors increased (Supplemental Figs 1 and 2).

## Discussion

In this retrospective cohort study of patients with LEAD after EVT, we discovered the following: (1) the co-presence of 3-MIA factors was significantly associated with both all-cause death and MACCE, (2) as MIA factors overlapped, the incidence of all-cause death and MACCE increased significantly, and (3) identical findings were observed in patients with CLTI.

Stenvinkel et al. [8] reported that malnutrition and inflammation are closely associated with atherosclerosis in end-stage renal disease, referred to as MIA syndrome. The MIA syndrome is associated with high mortality in patients [18]. We hypothesized that MIA syndrome worsens the prognosis of patients with LEAD, regardless of renal function. To the best of our knowledge, this is the first report to demonstrate an association between MIA syndrome, mortality, and MACCE in patients with LEAD who underwent EVT.

Nutritional status is an important factor in the prognosis of several diseases including LEAD [9, 10] and acute myocardial infarction [19]. Therefore, accurate assessment of nutritional status and appropriate management are crucial in improving the prognosis of patients with LEAD. BMI and serum albumin level are commonly used as indicators for nutritional assessment in routine clinical practice owing to their simplicity. However, these indicators alone may not accurately assess nutritional status as they are influenced by various factors such as inflammatory disease, additional weight gain due to fluid status, and liver and renal function [20–22]. Recently, the GNRI, an index calculated from BMI and serum albumin level [15], has been widely used for screening nutritional status because of its simplicity. Further, it may be useful in overcoming the shortcomings of single indicators such as serum albumin level and BMI. We previously reported that the GNRI is a useful prognostic predictor for patients with acute myocardial infarction [23] and stable CAD [24]. Moreover, Matsuo et al. [25] established that a low GNRI was an independent predictor of overall survival in patients with LEAD.

Theories describing the role of inflammation are well-established [5]. Several studies have reported that hs-CRP levels may predict future cardiovascular events and prognoses in patients with CAD, CVD or LEAD [11]. Furthermore, Sabatein et al. [26] demonstrated that hs-CRP (even 0.1–0.3 mg/dl, considered to be an intermediate risk factor) was a strong predictor of cardiovascular death, myocardial infarction, and stroke in patients with stable CAD.

It is a well-known polyvascular disease, in which atherosclerotic diseases often coexist in different vascular beds. Since the patients in this study had LEAD and underwent EVT, the “atherosclerotic” patients were defined as those with polyvascular disease including CAD and CVD in addition to LAED. In the REACH registry [27], 15.9% of patients with symptomatic atherosclerosis had polyvascular disease. The major cardiovascular event rates were significantly higher in patients with polyvascular disease than in those with monovascular disease or risk factors alone. Furthermore, a Fourier trial [12] stated that patients with LEAD and a history of myocardial infarction or stroke had significantly higher rates of cardiovascular death, myocardial infarction, and stroke than those without such a history.

In this study, we demonstrated that the co-presence of 3-MIA factors in patients with LEAD was significantly associated with all-cause death and MACCE. Furthermore, the incidence of all-cause death and MACCE increased significantly when these MIA factors overlapped.

These findings can be explained as follows. Inflammation plays a major role in the progression of atherosclerosis by disrupting vascular endothelial function, recruiting leukocytes, and inducing the expression of pro-inflammatory cytokines [5]. Inflammation, which is also a prominent factor in malnutrition, is associated with a higher fractional catabolic rate and a decreased synthesis rate of albumin. In addition, pro-inflammatory cytokines cause anorexia, reduced dietary protein intake, and further hypercatabolism [20]. Furthermore, albumin, a component of the GNRI, is an antioxidant, and malnutrition with low albumin levels increases oxidative stress, which may promote atherosclerosis [28]. Thus, malnutrition and inflammation form a vicious cascade that progresses to atherosclerosis and consequently worsens prognosis.

Dyslipidemia is well known as one of a risk factor for cardiovascular events [29]. However, several studies have revealed that lower level of total cholesterol or LDL-C were associated with an increased risk of all-cause death and MACCE among patients with AMI [30] or hemodialysis [31], which called “dyslipidemia paradox”. Furthermore, the paradox occurred in the group with malnutrition and inflammation, while a positive correlation between serum total cholesterol and the risk of all-cause death was observed in the group without malnutrition and inflammation [31]. Although the mechanism of the paradox remains uncertain, “dyslipidemia paradox” may be related to malnutrition and inflammation.

In the present study, as shown in Table 1, the frequency of dyslipidemia was significantly higher in SG and 3-MIA was significantly higher in DG. Malnutrition and inflammation are not only associated with atherosclerosis, but also with many other general conditions such as cellular immunity and frailty. Previous studies reported that malnutrition [3, 24, 25] and inflammation [6, 7, 11] are also risk factors for all-cause death and cardiovascular events. As the present study revealed, the presence of 3-MIA was a risk factor in patients with LEAD regardless of dyslipidemia, the classical risk factor. Therefore, malnutrition and inflammation may be uncontrollable residual risks with lipid-lowering therapy and further prospective studies are needed to explore the appropriate management of malnutrition and inflammation.

Due to the absence of data on changes in nutritional status over time, the impact of nutritional improvement on the clinical outcomes of patients with LEAD could not be determined. However, reports indicate that the GNRI in the remote phase of revascularization is associated with mortality risk, independent of baseline GNRI [9], and appropriate nutritional therapy may improve life expectancy. Feasible and effective nutritional therapy for patients with LEAD remain unestablished owing to the diverse comorbidities, swallowing functions, and preferences. Hence, further studies are required to establish appropriate nutritional therapies that may improve the prognosis of patients with LEAD.

In patients with stable CAD, low-dose colchicine, an anti-inflammatory agent, prevented cardiovascular events in a prospective clinical trial [32]. In addition, the CANTOS trial [33] revealed that suppression of inflammation by canakinumab, the IL-1β antibody, resulted in a 15% reduction of cardiovascular end points in patients with established atherosclerosis. The effect of anti-inflammatory therapy on the prognosis of patients with LEAD and the stratification of patient groups that benefit from anti-inflammatory therapy is unclear and further studies are warranted.

In the present study, as the MIA factors overlapped, the incidences of all-cause death and MACCE increased. In particular, 3-MIA was a crucial independent risk factor for all-cause mortality and MACCE in patients with LEAD after EVT. Therefore, risk stratification based on MIA factors may be useful in selecting patients who require more stringent risk factor management and may further lead to new treatment strategies for LEAD patients after EVT.

The present study had several limitations. First, this was a retrospective, single-center study with a relatively small cohort of patients. Second, nutritional status was assessed by employing GNRI, a composite index of BMI and serum albumin levels; however, we could not rule out systemic diseases such as subclinical malignancies that affect BMI and serum albumin levels. In addition, the GNRI was evaluated only once at the time of admission for EVT, and changes in the GNRI during the follow-up period were not evaluated. Third, we were unable to evaluate malnutrition status using other methods such as the Nutritional Risk Index, Mini Nutritional Assessment Short-Form scale, or Maastricht Index because the present study was retrospective, and we do not have the data required for those indexes. Fourth, we did not assess which nutritional therapy would improve the GNRI over time. Fifth, this study did not include patients with LEAD who were not suitable for EVT owing to their extremely poor general condition or advanced LEAD status.

In conclusion, the co-presence of the 3-MIA factors is a crucial risk factor for all-cause death and MACCE in patients with LEAD. Hence, risk stratification based on MIA factors may help identify patients with LEAD who require stringent management of risk factors and may lead to new treatment strategies.

## Supplementary Information

Below is the link to the electronic supplementary material.

Supplementary file1 (DOCX 464 KB)

## Data Availability

The datasets used and/or analyzed during the current study are available from the corresponding author on reasonable request.
