# Supplementary material for: Impact of co-presence of malnutrition-inflammation-atherosclerosis factors on prognosis in lower extremity artery disease patients after endovascular therapy
Source: Cardiovasc Interv Ther. 2024 Oct 24;40(1):102–11. doi: 10.1007/s12928-024-01058-6 (PMC11723883; doi:10.1007/s12928-024-01058-6)
Supplement: Supplementary file 1 — Supplementary file1 (DOCX 464 KB) [file 12928_2024_1058_MOESM1_ESM.docx]

**Supplemental material**

Impact of co-presence of malnutrition-inflammation-atherosclerosis factors on prognosis in lower extremity artery disease patients after endovascular therapy

Cardiovascular Intervention and Therapeutics

Kenta Ohmure, Daisuke Kanda, Yoshiyuki Ikeda, Akihiro Tokushige, Takeshi Sonoda, Ryo Arikawa, Kazuhiro Anzaki and Mitsuru Ohishi.

Corresponding author: Daisuke Kanda, MD, PhD, Assistant Professor

Department of Cardiovascular Medicine and Hypertension, Graduate School of Medical and Dental Sciences, Kagoshima University, Kagoshima, Japan.

E-mail: kanchan@m3.kufm.kagoshima-u.ac.jp

**Supplemental Table 1. Univariate cox proportional hazard analysis for all-cause death and MACCE in CLTI.**

|  | All-cause death | | | MACCE | | |
| --- | --- | --- | --- | --- | --- | --- |
| Variables | HR | 95%CI | *p* value | HR | 95%CI | *p* value |
| Age | 1.03 | 1.002–1.07 | 0.044 | 1.03 | 1.00–1.06 | 0.067 |
| Sex: male | 2.12 | 1.03–4.36 | 0.041 | 1.76 | 0.92–3.37 | 0.089 |
| Hypertension | 1.19 | 0.50–2.85 | 0.690 | 1.43 | 0.60–3.38 | 0.412 |
| Diabetes mellitus | 0.93 | 0.48–1.79 | 0.825 | 1.04 | 0.56–1.95 | 0.899 |
| Dyslipidemia | 0.89 | 0.48–1.68 | 0.723 | 0.85 | 0.47–1.55 | 0.603 |
| Current smoking | 0.83 | 0.29–2.33 | 0.718 | 1.16 | 0.49–2.74 | 0.740 |
| CKD | 11.53 | 1.58–84.04 | 0.016 | 4.37 | 1.35–14.15 | 0.014 |
| CCB | 0.62 | 0.33–1.18 | 0.147 | 0.57 | 0.31–1.05 | 0.072 |
| RAASi | 0.43 | 0.22–0.86 | 0.017 | 0.46 | 0.24–0.87 | 0.017 |
| β–blocker | 1.53 | 0.80–2.92 | 0.197 | 1.23 | 0.66–2.29 | 0.518 |
| Antiplatelet drug | 1.49 | 0.68–3.24 | 0.317 | 1.26 | 0.62–2.56 | 0.515 |
| Statin | 0.85 | 0.44–1.61 | 0.609 | 0.82 | 0.45–1.50 | 0.520 |
| Malnourished | 2.94 | 1.53–5.63 | 0.001 | 2.50 | 1.36–4.59 | 0.003 |
| Inflamed | 2.67 | 0.82–8.68 | 0.104 | 2.28 | 0.81–6.40 | 0.117 |
| Atherosclerotic | 1.91 | 0.97–3.78 | 0.063 | 1.97 | 1.03–3.77 | 0.040 |
| 3-MIA | 3.85 | 1.97–7.54 | <0.001 | 3.58 | 1.89–6.78 | <0.001 |
| LVEF | 0.98 | 0.96–1.01 | 0.166 | 0.99 | 0.97–1.01 | 0.287 |

CI, confidence interval; CCB, calcium channel blocker; CKD, chronic kidney disease; CLTI, chronic limb-threatening ischemia; HR, hazard ratio; LVEF, left ventricular ejection fraction; MACCE, major adverse cardiovascular and cerebrovascular events; 3-MIA, co-presence of three factors of malnutrition-inflammation-atherosclerosis syndrome (malnutrition, inflammation and atherosclerosis); RAASi, renin-angiotensin-aldosterone system inhibitor.

**Supplemental Table 2. Cox proportional hazards regression analysis models of 3-MIA for all-cause death and MACCE in CLTI.**

|  | All-cause death | | | MACCE | | |
| --- | --- | --- | --- | --- | --- | --- |
|  | HR | 95%CI | *p* value | HR | 95%CI | *p* value |
| Unadjusted | 3.85 | 1.97–7.54 | <0.001 | 3.58 | 1.89–6.78 | <0.001 |
| Model 1 | 4.18 | 2.07–8.45 | <0.001 | 3.75 | 1.94–7.26 | <0.001 |
| Model 2 | 4.01 | 1.98–8.11 | <0.001 | 3.63 | 1.83–7.03 | <0.001 |
| Model 3 | 4.08 | 2.01–8.28 | <0.001 | 3.65 | 1.88–7.10 | <0.001 |

Unadjusted, 3-MIA; Model 1, adjusted for age and sex (male); Model 2, adjusted for variables in Model 1 plus CKD; Model 3, adjusted for variables in Model 1 plus RAASi.

Abbreviations are listed in Supplemental Table 1.

**Supplemental Fig. 1. Kaplan–Meier analysis of survival rate based on number of MIA factors in CLTI.**


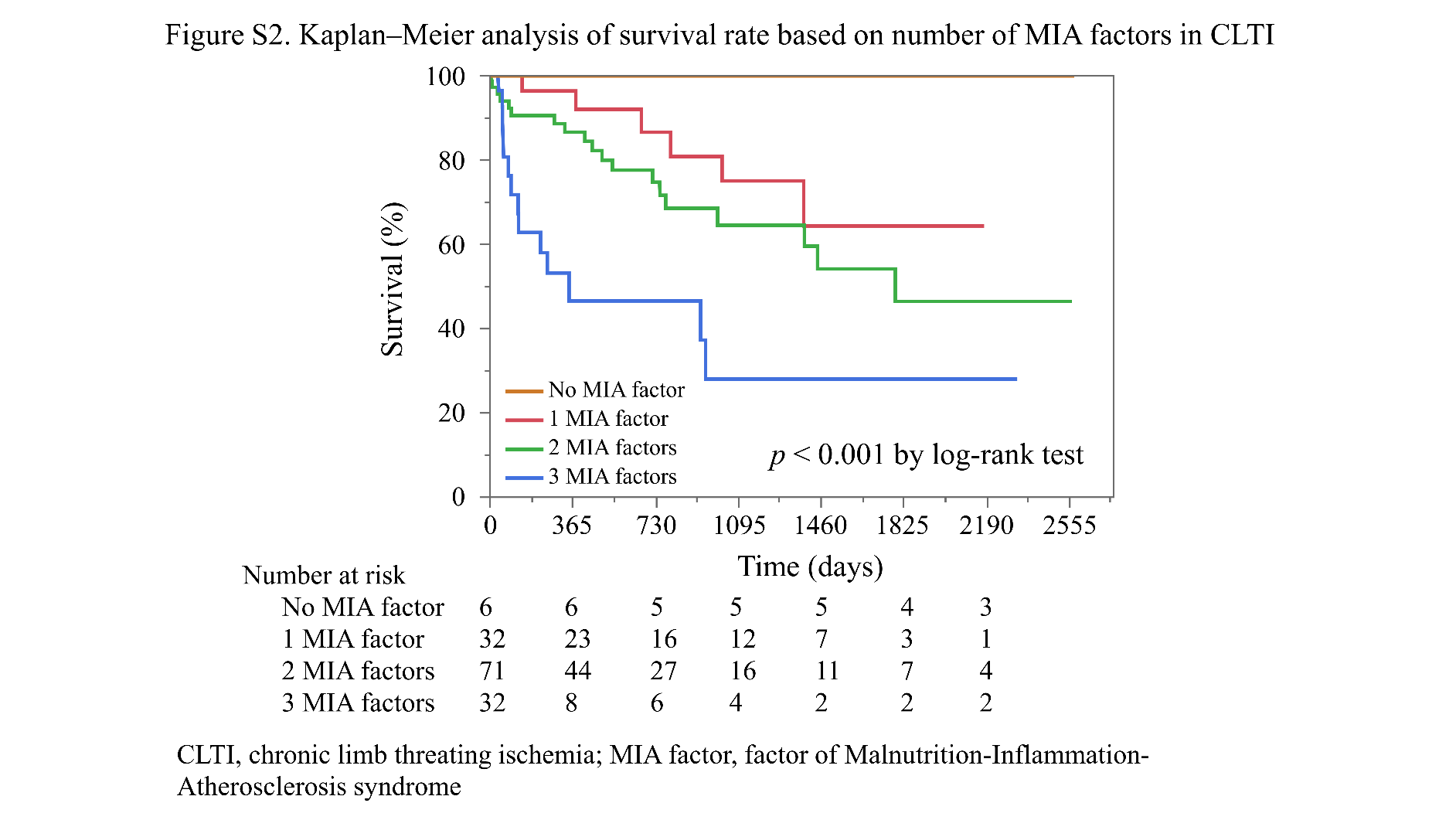


CLTI, chronic limb-threatening ischemia; MIA, malnutrition-inflammation-atherosclerosis syndrome.

**Supplemental Fig. 2. Cumulative incidences of MACCE based on number of MIA factors in CLTI.**


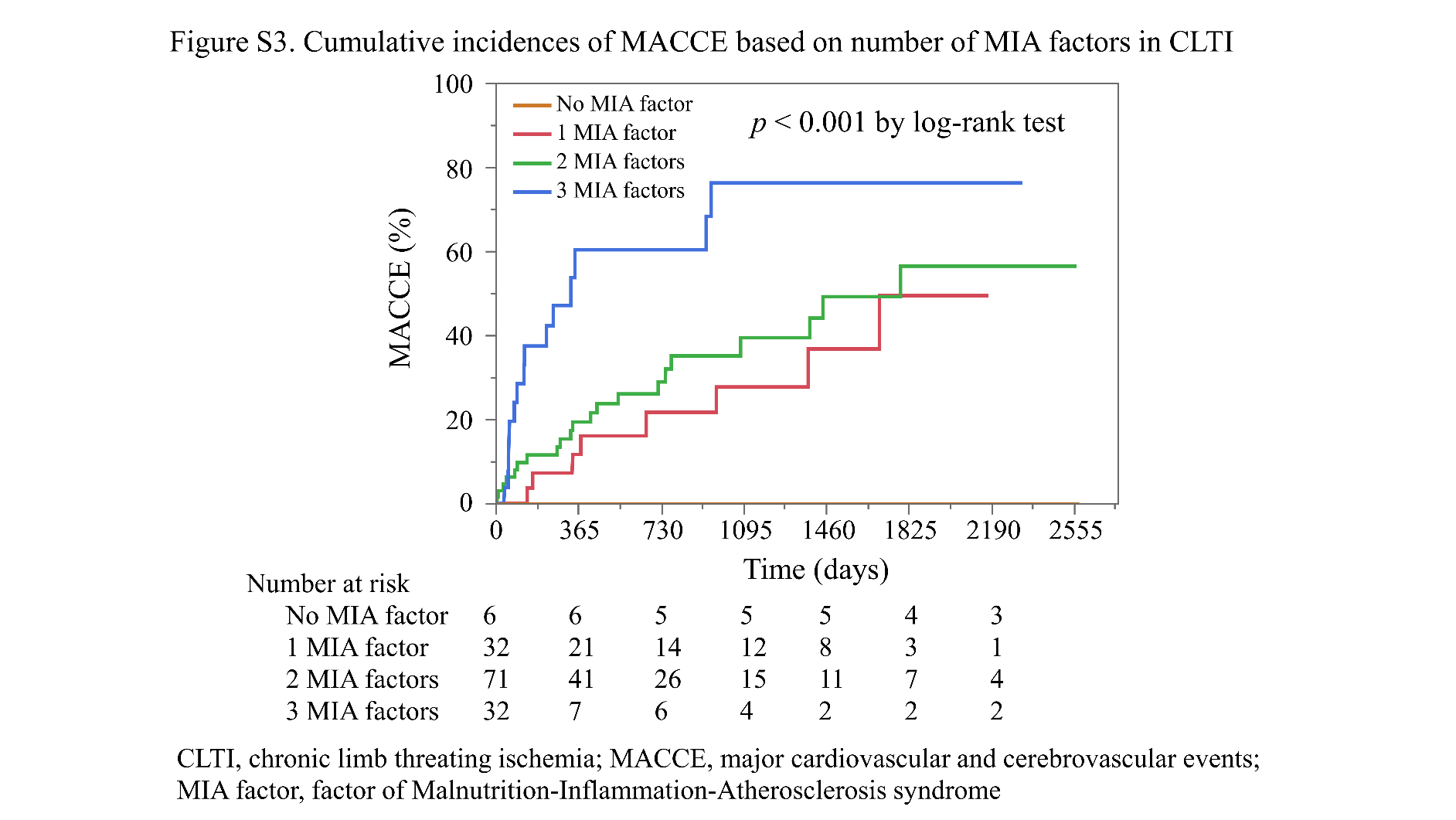


Abbreviations are listed in Supplemental Figure 1.
